# ^18^F-Fluorocholine PET and 4D-CT in Patients with Persistent and Recurrent Primary Hyperparathyroidism

**DOI:** 10.3390/diagnostics11122384

**Published:** 2021-12-17

**Authors:** Adrien Latge, Sophie Riehm, Michel Vix, Jacob Bani, Mihaela Ignat, Valentin Pretet, Mehdi Helali, Giorgio Treglia, Alessio Imperiale

**Affiliations:** 1 Department of Nuclear Medicine and Molecular Imaging, Institut de Cancérologie de Strasbourg Europe (ICANS), Strasbourg University Hospitals, 67033 Strasbourg, France; a.latge@icans.eu (A.L.); j.bani@icans.eu (J.B.); valentin.pretet@hotmail.com (V.P.); m.helali@icans.eu (M.H.); 2 Department of Radiology, Strasbourg University Hospitals, 67098 Strasbourg, France; sophie.riehm@chru-strasbourg.fr; 3 Department of General, Digestive, and Endocrine Surgery, IRCAD-IHU, Strasbourg University Hospitals, 67000 Strasbourg, France; michel.vix@chru-strasbourg.fr (M.V.); mihaela.ignat@chru-strasbourg.fr (M.I.); 4 Clinic for Nuclear Medicine, Imaging Institute of Southern Switzerland, Ente Ospedaliero Cantonale, 6500 Bellinzona, Switzerland; giorgio.treglia@eoc.ch; 5 Department of Nuclear Medicine and Molecular Imaging, Lausanne University Hospital, 1011 Lausanne, Switzerland; 6 Faculty of Biology and Medicine, University of Lausanne, 1011 Lausanne, Switzerland; 7 Faculty of Biomedical Sciences, Università Della Svizzera Italiana, 6900 Lugano, Switzerland; 8 Unité Mixte de Recherche 7178, Department of Radiobiology, Hadrontherapy and Molecular Imaging (DRHIM), Centre National de la Recherche Scientifique (CNRS), Institut Pluridisciplinaire Hubert Curien (IPHC), University of Strasbourg, 67000 Strasbourg, France

**Keywords:** primary hyperparathyroidism, persistent hyperparathyroidism, recurrent hyperparathyroidism, fluorocholine, PET, 4D-CT

## Abstract

Patients with primary hyperparathyroidism (pHPT) can develop persistent (P-pHPT) or recurrent (R-pHPT) disease after parathyroidectomy. Before recommending reoperation, recurrence must be accurately identified because of the high risk of complications. Our study evaluates ^18^F-fluorocholine (^18^F-FCH) PET/CT and 4D-CT integrated in PET/4D-CT in patients with P-pHPT/R-pHPT. Patients with P-pHPT/R-pHPT investigated by ^18^F-FCH PET/4D-CT between May 2018 and March 2021 were retrospectively included. Forty-two patients were included, 37 of whom underwent 4D-CT. The sensitivity and detection rate (DR%) were 95% and 88% for ^18^F-FCH PET/CT and 70% and 63% for 4D-CT, respectively. PET/CT and 4D-CT were concordant in 18/24 glands and concordant and positive in 15/24 (63%) glands. Discordant results were obtained for 6/24 glands. The surgical success rate was 65%. PET/CT showed significantly higher sensitivity than 4D-CT. Dynamic CT allowed the identification of no additional glands missed by PET/CT, and the combination of the 2 techniques did not improve the sensitivity or DR%. ^18^F-FCH PET/CT appears to be a valuable technique to accurately detect hyperfunctioning parathyroid tissue in patients with P-pHPT/R-pHPT and is better than 4D-CT. Except for cases with doubtful locations of PET targets that may require 4D-CT for surgical guidance, standard nonenhanced ^18^F-FCH PET/CT can be effectively recommended in patients with P-pHPT/R-pHPT before reoperation.

## 1. Introduction

Primary hyperparathyroidism (pHPT) is an endocrine disease related to excessive secretion of parathyroid hormone (PTH) from one or more hyperfunctioning parathyroid glands. Surgical removal of pathological parathyroid prevents severe hypercalcemia-related complications and remains the sole treatment with curative intent in symptomatic patients [1,2]. A safe and successful surgical approach for pHPT depends on the surgeon’s ability to accurately identify all abnormal parathyroid glands [3]. Otherwise, long-term persistent disease can occur. While most patients with pHPT initially undergo curative surgery, approximately 2–10% of cases develop persistent or recurrent disease after initial surgery [4,5,6]. Persistent primary hyperparathyroidism (P-pHPT) is diagnosed in the early postoperative period and is defined by a persistent high calcium serum concentration and/or elevated or inappropriately normal serum PTH values within six months after parathyroid surgery. However, if both hypercalcemia and/or elevated PTH values recur six months after normalization of serum calcium after surgery, the diagnosis of recurrent primary hyperparathyroidism (R-pHPT) is retained [7]. Surgeon expertise and/or a low hospital volume, patients over 70 years old, and inconclusive parathyroid scintigraphy are considered predictive factors of P-pHPT and R-pHPT [8].

In contrast to initial neck surgery, reoperation for P-pHPT/R-pHPT must be evaluated very carefully due to a higher rate of complications than the initial surgery. The risk of transient or permanent recurrent laryngeal nerve palsy is considerably higher, as permanent hypoparathyroidism results in long-lasting hypocalcemia. To improve the chances of success of repeated surgery, the site(s) of recurrence must be accurately identified before reoperation is recommended [9]. The decision toward reoperation should be made with care, even for experienced teams [10,11]. If the pathological gland(s) cannot be identified in advance, the rationale of reoperation is doubtful, and medical treatment with calcimimetics should be considered. Therefore, a diagnostic technique with high sensitivity is needed to reduce the number of secreting parathyroids that may be undetected, resulting in P-pHPT/R-pHPT. Typical situations are the presence of unknown ectopic glands [12], unrecognized multigland disease [9], and a negative preoperative imaging workup [13].

Preoperative detection of multigland disease and the location of the hyperfunctioning parathyroid gland in patients with P-pHPT/R-pHPT remain challenging. Usually, a combination of various preoperative imaging techniques is required to improve patient outcomes. Although ^99m^Tc-sestamibi parathyroid scintigraphy and neck ultrasound are often used as first-line imaging, their diagnostic performance is influenced by the type of scintigraphy and imaging protocol (^99m^Tc-sestamibi scintigraphy, ^123^I/^99m^Tc-sestamib dual-isotope scintigraphy with the subtraction technique, tomographic vs. planar acquisition, pinhole collimator acquisition) and by the experience of the operator, particularly in patients with previous neck surgery [14,15].

Four-dimensional computed tomography (4D-CT) uses the dynamics of contrast enhancement (nonenhanced phase, followed by contrast-enhanced arterial and venous CT phases) to identify abnormal parathyroid glands [16]. In the reoperative setting, 4D-CT sensitivity varies from 50 to 93% [5,17,18], with a high radiation burden to the neck and upper thorax. A growing body of data highlights the high sensitivity of ^18^F-fluorocholine (^18^F-FCH) positron emission tomography/computed tomography (PET/CT) to detect hyperfunctioning parathyroids (sensitivity and a detection rate up to 90% and 80%, respectively) in patients with pHPT [19]. Thus, when available, ^18^F-FCH PET/CT should be proposed in patients with pHPT due to its excellent diagnostic performance and reasonable patient radiation exposure [20]. In the future, ^18^F-FCH PET/CT could also be considered an ideal candidate to replace neck ultrasound and parathyroid scintigraphy for preoperative localization of pathological parathyroid tissue in patients with pHPT [20].

At present, the value of ^18^F-FCH PET/CT in problematic cases of postoperative P-pHPT/R-pHPT remains largely unknown and has only occasionally been analyzed. Only limited data are available regarding the promising role of ^18^F-FCH PET/CT in the reoperative setting [21,22], especially when combined with contrast-enhanced CT. More evidence is needed to improve the current knowledge about the diagnostic role of ^18^F-FCH PET/CT in this challenging clinical scenario. Thus, the primary aim of the present study was to evaluate and compare the sensitivity and detection rate (DR%) of ^18^F-FCH PET and 4D-CT in patients with P-pHPT/R-pHPT examined by ^18^F-FCH PET/4D-CT.

## 2. Materials and Methods

### 2.1. Patient Population

This is a monocentric, retrospective, noninterventional study including patients with pHPT addressed to our institution for ^18^F-FCH PET/CT investigation between May 2018 and March 2021. Only patients with P-pHPT/R-pHPT and available clinical and biological follow-up data after PET/4D-CT were selected and included in the analysis. The following patient clinical, imaging and biological data were retrieved from hospital databases, clinician reports, and biomedical laboratories: sex, age, previous parathyroid surgery, the results of diagnostic imaging performed before PET/CT (i.e., neck ultrasound (US), parathyroid scintigraphy, neck CT or magnetic resonance imaging (MRI)), patient genetic status, calcimimetic treatment, hypercalcemia-related symptoms, and PTH, calcium, phosphate and 25-OH vitamin D serum concentrations. Surgical procedures, perioperative PTH measurements, and pathological reports concerning the second parathyroid surgery were collected for patients who underwent reoperation after ^18^F-FCH PET/CT.

A cross-disciplinary team determined ^18^F-FCH PET/4D-CT indications. In accordance with local institutional guidelines, all patients included provided free and written informed consent for the use of anonymous personal medical data extracted from their files for scientific or epidemiological purposes. The local Institutional Review Board approved this retrospective study (CE-2021-104).

### 2.2. ^18^F-FCH PET/4D-CT Technical Procedures and Interpretation

All exams were performed using integrated Biograph Vision PET/128-slice CT system (Siemens Healthcare, Erlanger, Germany) equipped with time-of-flight measurement capacity; 10-min PET acquisition from the mandible to the carina was performed in the supine position with arms along the body and a headrest at approximately 60 min after intravenous injection of 3–3.5 MBq/kg of ^18^F-FCH. PET datasets were reconstructed iteratively using no contrast-enhanced CT for attenuation correction. PET data were also corrected for scattering, random coincidences and radioactive decay.

In patients without contraindications, a dynamic four-phase CT scan was performed. 4D-CT included nonenhanced CT (140 kV, 115 mA, 1 s per rotation and pitch 0.8, slice thickness of 1 mm) followed by arterial phase (10–15 s after injection, aortic arch threshold >80 HU), venous phase (45 s after injection), and late-venous phase (70 s after injection) acquisition; 75 mL of iodine contrast agent (Iomeron 400 mg/mL, Bracco Imaging, Milan, Italy) was intravenously injected with a 2.5–3 mL/s flow rate followed by a saline chaser. The CT parameters of arterial and venous phases were: 120 kV, 1 and 15 mAs, 1-s rotation time, pitch 0.8, and a slice thickness of 1 mm. CARE Dose4D (Siemens Healthcare, Erlanger, Germany) combined with sinogram-affirmed iterative reconstruction (SAFIRE, Siemens Healthcare, Erlanger, Germany) was used. Diabetic patients withdrew metformin treatment for 2 days after PET/4D-CT, and abundant hydration was recommended.

^18^F-FCH PET/(nonenhanced)CT and 4D-CT were independently interpreted on a dedicated workstation by one expert nuclear medicine physician and one radiologist, respectively. Referring physicians were aware of the patient history, clinical features, and biological data and previous parathyroid imaging results but were blinded to the results of either PET or 4D-CT. ^18^F-FCH PET and 4D-CT were qualitatively interpreted as positive or negative. Focal nonphysiological uptake corresponding to any cervical or thoracic nodule discriminable from thyroid tissue, positioned in typical parathyroid sites or in ectopic areas, was considered positive on ^18^F-FCH PET. For each positive gland, the maximum standardized uptake value (SUVmax) was assessed. Focal cervical or thoracic nodules exhibiting different densities compared to thyroid tissue and dynamic patterns of contrast media enhancement compatible with hyperfunctioning parathyroid glands were considered positive on 4D-CT after radiologist validation in regard to varied enhancement patterns [23]. The number of pathological findings on PET/CT and 4D-CT, topography in reference to the midline and thyroid gland and ectopic locations were recorded.

### 2.3. Gold Standard

In patients who underwent parathyroid surgery after ^18^F-FCH PET/CT, histopathology associated with normalization of PTH serum levels was considered the diagnostic gold standard. In these patients, PTH and serum calcium values during the follow-up were used to differentiate cured from uncured patients as follows: (i) cured: patients without biological abnormalities at least 6 months after surgery (mean follow-up: 12.1 months); (ii) uncured: patients with P-pHPT appearing in the following months after surgery (mean follow-up: 10.6 months). Persistent or recurrent abnormal serum PTH (>65 pg/mL) and calcium levels (>2.6 mmol/L) were considered indicative of persistent disease (uncured).

In nonoperated patients, biological data during the follow-up were used as the gold standard: patients with elevated serum PTH (>65 pg/mL) without 25-OH vitamin D deficiency (>30 ng/mL) or hypercalcemia (>2.6 mmol/L) with normal or elevated serum PTH (>10 pg/mL) were considered positive for the presence of hyperfunctioning parathyroid tissue (one or more hyperfunctioning parathyroids).

### 2.4. Statistical Analysis

The results for continuous data are expressed as the mean ± standard deviation and the range. Categorical variables are presented as numbers and percentages. The sensitivity, specificity, positive and negative predictive values (PPV and NPV) and detection rate (DR%) of ^18^F-FCH PET/CT and 4D-CT were calculated per patient and per lesion analysis.

Regarding the per lesion analysis, sensitivity was evaluated on surgically treated patients, while DR% was assessed in the overall population. Diagnostic performance was determined separately for (1) identification on the correct side (left/right) and (2) the gland embryological origin using surgical findings as the standard of reference. ^18^F-FCH PET/CT and 4D-CT results were compared using the McNemar test. Differences between groups were assessed by Student’s *t*-test for continuous variables. Two-sided *p* values below 0.05 were considered significant. Statistical analyses were performed using open access statistical software (biostatgv.sentiweb.fr, Institut Pierre Louis d’Epidémiologie et de Santé Publique, UMR S 1136, INSERM—Sorbonne Université, Paris, France).

## 3. Results

### 3.1. Patient Population

From May 2018 to March 2021, 47 patients examined by ^18^F-FCH PET/CT were retrieved from our institutional database. Five patients were excluded because of missing follow-up data. Thus, 42 patients (38 women) presenting with either P-pHPT (25/42, 60%) or R-pHPT (17/42, 40%) were finally enrolled. The mean age was 58 ± 15 years (range: 15–83). Among the included patients, 15/42 (36%) suffered from HPT-related complications, including bone demineralization, urinary lithiasis or hypercalcemia-related pancreatitis. The mean PTH and calcium concentrations at the time of ^18^F-FCH PET/CT were 118 pg/mL (range: 15–746) and 2.54 mmol/L (range: 2.22–3.05), respectively; 4/42 (10%) patients had multiple endocrine neoplasia type-1 (MEN-1), and 1/42 (2%) had familial hypocalciuric hypercalcemia (FHH). At the time of ^18^F-FCH PET/CT, 6/42 (14%) patients were treated with cinacalcet. Patient characteristics are summarized in Table 1, whereas characteristics of reoperated patients are summarized in Table 2.

In the month following ^18^F-FCH PET/4D-CT, 20/42 (48%) patients were scheduled for reoperation and underwent exploratory cervicotomy (16/20, 80%) or minimally invasive parathyroidectomy (MIP) (3/20, 15%). The surgical procedure was not available for one patient.

The surgical success rate was 60% (12/20 patients). Overall, 22 pathological parathyroids were localized and excised. One or multiple parathyroid adenomas were histologically confirmed in 9/20 (45%) patients, and parathyroid hyperplasia was confirmed in 6/20 (30%) patients. Adenoma was indistinguishable from hyperplasia in 2/20 (10%) patients. In 4 patients, pathology failed to show parathyroid tissue and revealed one benign ganglioneuroma and 3 inflammatory lymph nodes. In reoperated patients, the mean preoperative and postoperative PTH levels were 124 ± 69 pg/mL and 39 ± 38 pg/mL, respectively.

All 42 patients (100%) underwent ^18^F-FCH PET/CT, and 37/42 (88%) underwent 4D-CT (5 patients had a previous history of allergic reaction to iodine contrast media or impaired renal function contraindicating enhanced CT); 16/42 (38%), 14/42 (33%), and 8/42 (19%) patients underwent neck US, parathyroid scintigraphy, or both, respectively, before PET/CT.

### 3.2. ^18^F-FCH PET/CT Results

According to a per patient-based analysis performed on the overall population, ^18^F-FCH PET/CT was true-positive in 28/42 patients (67%), true-negative in 4/42 patients (10%), false-negative in 9/42 patients (21%), and false-positive in the remaining case (2%). The resulting sensitivity, specificity, PPV, NPV, and DR% were 76%, 80%, 97%, 31%, and 69%, respectively.

A per lesion-based analysis was performed only on the 20 reoperated patients (Table 3). According to the pathological examination of 26 tissue specimens available after neck surgery, ^18^F-FCH PET/CT was true-positive in 21 cases (80%), false-positive in 2 cases (8%, one ganglioneuroma and one lymph node), true-negative in 2 cases (8%, 2 lymph nodes), and false-negative in one case (4%, one hyperplasic gland). ^18^F-FCH PET/CT sensitivity, specificity, PPV, NPV, and DR% were 95%, 50%, 91%, 67%, and 88%, respectively.

PET/CT correctly lateralized all 22 detected pathological parathyroids (left/right side) and identified the embryological origin in 20/22 (91%) glands (one gland was wrongly classified as a lower instead of an upper gland, and 1 was classified as of uncertain origin).

The mean SUVmax of hyperfunctioning parathyroids was 5.2 ± 2.3. The 36 adenomas showed a higher mean SUVmax (6.1 ± 3.4) than the 17 hyperplasic glands (4.7 ± 1.4), without reaching statistical significance. Regarding the possible interference of calcimimetic treatment in the ability of PET to detect hyperfunctioning parathyroids, only 1 of 6 patients treated with cinacalcet at the time of examination showed negative PET results. In our cohort, 16/42 patients (38%) and 14/47 patients (30%) underwent neck US and parathyroid scintigraphy, respectively (performed with different acquisition protocols), before ^18^F-FCH PET/CT, precluding meaningful statistical analysis. Considering this limitation, the sensitivity and DR% appeared higher for PET/CT than for both US and scintigraphy. Differences were not always statistically significant, mainly due to the limited number of analyzed patients and glands (Table 3).

### 3.3. 4D-CT Results

**Figure 1:**
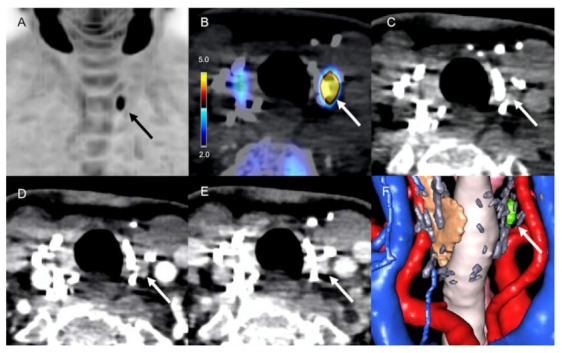
^18^F-FCH PET/CT, 4D-CT and 3D-virtual neck exploration of a 74-year-old woman with recurrent pHPT and a history of left thyroidectomy and removal of the upper right hyperplasic parathyroid gland. From left to right, top to bottom: lower left adenoma (black and white arrows) with high focal accumulation of 18F-FCH ((**A**) anterior view of maximum intensity projection, (**B**) axial PET/CT image, SUVmax = 9.67), spontaneous density of 71 HU (**C**) no contrast enhancement on arterial-phase CT ((**D**) density of 71 HU), delayed enhancement at the late venous phase ((**E**) density of 140 HU), 3D-virtual neck exploration (**F**).

In the per patient-based analysis performed on the overall population, 14/37 cases (38%) examined by 4D-CT were true-positive, 3/37 cases (8%) were true-negative, 19/37 cases (51%) were false-negative, and one case was false-positive (3%). Thus, the 4D-CT sensitivity, specificity, PPV, NPV, and DR% were 42%, 75%, 93%, 14%, and 41%, respectively. According to the per lesion-based analysis performed on the 18/20 reoperated patients who performed 4D-CT, the sensitivity, specificity, PPV, NPV, and DR% were 70%, 75%, 93%, 33%, and 63%, respectively (Table 2). According to the results of the pathological examination of 24 tissular specimens obtained after neck surgery (Table 3), 4D-CT was true-positive in 14/24 cases (58.4%), false-positive in 1/24 cases (4.2%, 1 ganglioneuroma), true-negative in 3/24 cases (12.5%, 2 lymph nodes, one thymic tissue specimen), and false-negative in 6/24 cases (25%, 3 adenomas, 3 hyperplasic glands) (Figure 1).

### 3.4. ^18^F-FCH PET/CT versus 4D-CT

**Figure 2:**
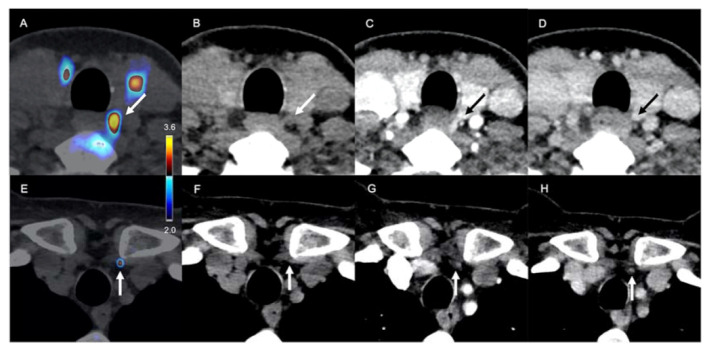
^18^F-FCH PET/CT and 4D-CT of a 56-year-old woman with recurrent pHPT and a history of upper and lower right parathyroidectomy (adenoma and hyperplasic gland, respectively). From left to right: fused PET/CT, non-contrast-enhanced CT, arterial-phase CT (30 s), venous-phase CT (70 s). Upper panel: lateral esophageal upper left adenoma (white and black arrows) with focal uptake of ^18^F-FCH (SUVmax = 4) (**A**), spontaneous density of 66 HU (**B**), high enhancement at the arterial phase (137 HU) (**C**), and washout > 20 HU at the venous phase (102 HU) (**D**). Lower panel: hyperfunctioning parathyroid in the left thyrothymic ligament (white arrows) with focal uptake of ^18^F-FCH (SUVmax = 3.2) (**E**), spontaneous density of 50 HU (**F**), no significant enhancement at the arterial phase (62 HU) (**G**) or venous phase (49 HU) (**H**).

Thirty-seven patients underwent integrated ^18^F-FCH PET/4D-CT (Table 2) and were considered for direct comparison of ^18^F-FCH PET and 4D-CT. PET/CT and 4D-CT were concordant in 18/24 (75%) glands and concordant positive in 15/24 (63%) glands. Discordant results (PET+/4D-CT-) were obtained for 6/24 glands (25%) (Figure 2).

In the per lesion-based analysis, PET/CT showed a significantly higher sensitivity (95% vs. 70%, *p* = 0.04) and DR% (88% vs. 63, *p* = 0.04) than 4D-CT. The DR% was also higher for PET/CT than 4D-CT in the per patient analysis (70% vs. 41%, *p* < 0.01) (Table 2). Overall, in our cohort, a modest impact of dynamic multiphase CT integrated into PET/4D-CT on lesion detection was noted. ^18^F-FCH PET/CT and 4D-CT interpretation was discordant for 5/6 orthotopic pathological parathyroids, all of which were true-positive on PET/CT and false-negative on 4D-CT (mean weight: 0.2 g, mean volume: 0.69 cm^3^). The remaining lesion was a mediastinal lymph node classified as false-positive by PET/CT and true-negative by 4D-CT.

## 4. Discussion

Our findings consolidate the diagnostic role of ^18^F-FCH PET/CT in challenging patients with P-pHPT/R-pHPT after initial parathyroidectomy, appearing more effective than dynamic 4D-CT in this specific clinical situation.

^18^F-FCH PET/CT was demonstrated to be an excellent diagnostic tool with similar performance in terms of lesion-based sensitivity and the DR% to that reported in patients without previous parathyroid surgery [19,24]. In addition, we provide further data to enrich the debate about the role of 4D-CT integrated into a single ^18^F-FCH PET/4D-CT device.

To our knowledge, only two recent studies specifically address the value of ^18^F-FCH PET/4D-CT in patients with pHPT and previous neck surgery (mostly related to parathyroid ablation) [21,22]. Christakis et al. [22] reported the first UK prospective cohort of 12 patients with P-pHPT (*n* = 11) and R-pHPT (*n* = 1) who had at least one previous failed parathyroid operation and negative US and parathyroid scintigraphy postoperatively. Arterial-enhanced CT of the neck followed by portal venous CT for the attenuation correction protocol was integrated into the PET/CT study. Any positive or doubtful lesion identified on PET/CT led to surgical resection. Seven patients were cured, including 3 with ectopic localizations. Five patients had persistent disease after repeat surgery, with false-positive lesions on PET images (lymph nodes). The arterial enhancement of the possible target on ^18^F-FCH PET was different between the cured/noncured patients (*p* = 0.007). Accordingly, the combination of PET and arterial enhanced CT may be useful to guide the identification of small potential adenomas (by high PET sensitivity), reducing the rate of nodal false-positive results (by high arterial enhanced CT specificity). Moreover, the proposed 2-phase CT protocol seems to be an acceptable compromise allowing diagnostic accuracy to reduce patient radiation exposure. In the same year, Amadou et al. [21] retrospectively investigated ^18^F-FCH PET/CT and 4D-CT to guide second surgery in 29 patients with pHPT and previous parathyroid (*n* = 23, 79%) or thyroid surgery (*n* = 6, 21%). ^18^F-FCH PET/CT and 4D-CT had sensitivities of 96% and 75%, respectively. However, 4D-CT showed higher specificity than ^18^F-FCH PET/CT. Indeed, 4D-CT could be used as a valuable confirmatory imaging modality in discordant cases to optimize the surgical strategy. Moreover, this study confirms the superiority of both ^18^F-FCH PET/CT and 4D-CT compared to first-line imaging (US and parathyroid scintigraphy) in this challenging population. Interestingly, US performance increased when performed after ^18^F-FCH PET/CT in an open reading.

Despite a significant cure rate (60%), in our population, five patients were not cured after successful removal of one hyperfunctioning parathyroid gland, demonstrating the high rate of subclinical multiglandular disease in this challenging population previously treated by parathyroid surgery. One of the main reasons for initial operative failure may be the presence of four-gland hyperplasia (FGH) [12]. Similarly, available data suggest that patients with double adenomas are more likely to present P-pHPT/R-pHPT after the first parathyroid surgery. In our cohort, 8/42 (19%) patients had a history of operated dual autonomous glands, and 3 (8%) had previous subtotal parathyroidectomy for FGH. The high rate of patients with multiple hyperfunctioning parathyroids [6,25,26] warrants rigorous intraoperative examination and an attentive follow-up after parathyroidectomy [27].

The added value of 4D-CT integrated into an ^18^F-FCH PET/4D-CT study was first described by Piccardo et al. [28] in 2018, who investigated 44 patients with pHPT. Both the DR% (72.7% vs. 56.8%) and sensitivity (100% vs. 80%) were significantly higher for PET/4D-CT than for nonenhanced PET/CT, emphasizing the positive value of enhanced CT. These findings conflict with our results in both the general pHPT population [29] and in patients with P-pHPT/R-pHPT, showing that the combination of both techniques did not significantly improve diagnostic sensitivity.

Notably, a recent meta-analysis [30] mainly focusing on cases without P-pHPT/R-pHPT seems to confirm our findings in patients with recurrent/persistent disease, suggesting that the sensitivity of ^18^F-FCH PET/CT and ^18^F-FCH PET/4D-CT is higher than that of 4D-CT, but only a minor difference was reported between ^18^F-FCH PET/CT and ^18^F-FCH PET/4D-CT.

Several explanations may account for the suboptimal diagnostic performance of 4D-CT [30]. Overall, the identification of hyperfunctioning parathyroid remnants or recurrence can be difficult, mainly due to postoperative local morphological changes related to persisting neck fibrosis. The iatrogenic modification of the regional anatomic architecture may be responsible for alteration of the typical pattern of tissue enhancement after contrast i.v. injection. Thus, the arrival of contrast media during the early arterial phase may be delayed and less pronounced due to scar formation. This phenomenon is potentially responsible for misinformed interpretations, particularly in patients with a history of bilateral cervicotomy. On the other hand, the analysis of functional images as ^18^F-FCH PET is only slightly influenced by posttreatment sequelae in patients with persistent/recurrent disease. Another cause of reduced 4D-CT accuracy may be related to the altered flow of the iodinated contrast agent during its passage through the subclavian vein, potentially causing scatter artifacts in the thyroid region and thus leading to potential erroneous interpretations, which is often the case in arterial-phase CT (30 s), complicating the quantitative evaluation of target washout during delayed phases at 45 s and 70 s after contrast media administration. The presence of surgical clips after previous neck surgery is also a confounding factor, with possible metallic artifacts hindering local recurrence (Figure 1).

Our study suffers from several limitations requiring commentary. First, the retrospective nature resulted in the inclusion of a limited number of patients. Second, approximately one-half of the included patients did not undergo reoperation after imaging revaluation. Patients’ refusal of new surgery, absence of symptomatology, normocalcemia with isolated elevated PTH, or postponed surgery due to the COVID-19 pandemic mainly explain the low rate of reoperated patients. Finally, neck US and parathyroid scintigraphy were not performed in all patients before PET, precluding robust comparison among diagnostic techniques and potentially introducing a patient selection bias. However, in our tertiary referral surgical center, we faced an important variability in the diagnostic precision of the imaging studies that patients had already obtained before referral. Our practice is not to repeat imaging to avoid supplementary radiation, costs and delays. This issue especially concerns neck US, which suffers from important heterogeneity in terms of the quality of the radiologist’s interpretation, and parathyroid scintigraphy, which is usually performed with different imaging protocols.

MRI provides good soft tissue contrast and could provide better differentiation than CT between parathyroid, muscle, and other soft tissues as lymphatic structures. Moreover, no exposure to radiation is required during an MRI procedure [15]. Thus, ^18^F-FCH PET/MR may be the next step in assessing pHPT [24,31,32], but it is still too early to define its benefit compared to ^18^F-FCH PET/CT, particularly in the subgroup of patients with P-pHPT/R-pHPT.

## 5. Conclusions

In conclusion, ^18^F-FCH PET/CT appears to be a valuable technique to accurately detect hyperfunctioning parathyroids in patients with P-pHPT/R-pHPT and is better than 4D-CT, without increasing patient radiation exposure due to repeated CT acquisitions. Except for cases with doubtful locations of functional targets that may require 4D-CT for surgical guidance, standard nonenhanced ^18^F-FCH PET/CT can be effectively recommended in patients with P-pHPT/R-pHPT before reoperation. The real surgical impact of these findings in patients with persistent/recurrent disease should be assessed in prospective multicenter clinical trials.

## Figures and Tables

**Table 1:** Overview of the clinical and biological characteristics of the included patients.

Criteria	Number
Sex (%)	*n* = 42
Female/Male	38 (90.5%)/4 (9.5%)
Age (years, mean)	58.4
Localization of parathyroid gland removed during prior surgery (%)	*n* = 55
Upper right/Lower right	15 (27.3%)/15 (27.3%)
Upper left/Lower left	13 (23.6%)/10 (18.2%)
Unknown localization	2 (3.6%)
Number of removed glands per patient during prior surgery (%)	*n* = 42
1 gland	23 (54.8%)
2 glands	11 (26.2%)
3 glands	2 (4.7%)
Subtotal parathyroidectomy	1 (2.4%)
No glands removed	5 (11.9%)
Pathology after first surgery	*n* = 55
Adenoma	34 (61.8%)
Hyperplasia	13 (23.6%)
Hypercellular (adenoma or hyperplasia)	3 (5.5%)
Normal gland	4 (7.3%)
Unknown	1 (1.8%)
Type of pHPT (%)	*n* = 42
Persistent pHPT/Reccurent p-HPT	25 (59.5%)/17 (40.5%)
Biological features (mean ± SD)	
PTH (pg/mL)	118 ± 114
Calcemia (mmol/L)	2.54 ± 0.18
Phosphatemia (mmol/L)	0.95 ± 0.18
25-OH Vitamin D (ng/mL)	30.4 ± 11.2
Patient clinical symptomatology (%)	*n* = 42
None or undocumented	27 (64.3%)
Osteroporosis/osteopenia	4 (9.5%)
Urinary lithiasis/renal colic	9 (21.4%)
Pancreatitis	2 (4.8%)
Genetical status (%)	*n* = 42
No mutation or undocumented	37 (88.1%)
MEN-1	4 (9.5%)
FHH	1 (2.4%)
Cinacalcet treatment (%)	*n* = 42
Yes/No	6 (14.3%)/36 (85.7%)

MEN-1: Multiple endocrine neoplasia type-1, FHH: Familial hypocalciuric hypercalcemia.

**Table 2:** Imaging, pathology and PTH values after second parathyroidectomy in reoperated patients after ^18^F-FCH PET/4D-CT.

Patient	Sex	Age (Years)	HPT Type	Genetic Status	Cinacalcet	US	^99m^Tc-Sestamibi	^18^F-FCH PET/CT	4D-CT	Gland(s) Localization(s)	Histology (From PET Target)	Volume (cm^3^)	Weight (g)	Surgical Technique	Pre-Operative PTH (pg/mL)	Post-Operative PTH (pg/mL)	Patient Cured
1	F	61	P		No		+/−/+	+/+/+	−/+/+	UR/LR/LL	3 Hpl	2.0/0.8/0.8	nc	CE	80.5	8.3	Yes
2	F	73	R		Yes	−	−	+	−	UL	Ad	0.4	0.3	CE	145.8	6.2	Yes
3	F	52	R		No	+	−	+	+	UL	Ad	0.1	<0.1	MIP	100.9	10.8	Yes
4	M	65	R		Yes			+		ectopic	Hpl	0.7	nc	MIP	155.5	110.2	No
5	F	78	R		No		−	+	−	LR	Hpl	0.3	<0.1	CE	90.2	11.7	Yes
6	F	15	P	MEN-1	No			+	+	UR	Ad or Hpl	0.5	0.3	CE	127.1	14.7	Yes
7	F	71	P		Yes	−		+	+	LL	Ad	0.8	nc	CE	80	47	Yes
8	F	68	R		No	+	−	+	+	UR	Ganglioneuroma	nc	nc	CE	112	55.9	No
9	F	28	P	MEN-1	No		−/+	+/−/+	+/−/+	LR/UL/UL	3 Hpl	nc	nc	CE	nc	nc	Yes
10	F	54	R		Yes	+	−	+	+	UR	Ad	1.1	0.6	CE	146.3	8.7	Yes
11	F	69	P		No		+	+	+	LL	Ad	0.4	0.2	CE	69.9	11.3	Yes
12	F	69	P		No			−	−		Lymph node	nc	nc	CE	80	131	No
13	F	64	P		No			+	+	LL	Hpl	0.2	<0.1	CE	75.4	23.3	No
14	F	60	P		No			+/+	−/+	LR/UL	2 Ad	0.4/1.0	0.2/0.5	CE	158	43	Yes
15	F	28	R	MEN-1	No	+		+	+	LR	Ad	fragmented	0.4	CE	330.4	31.4	No *
16	M	41	R	MEN-1	No	−	+	+		LR	Ad or Hpl	0.8	0.2	MIP	68.7	15.7	No
17	F	56	R		No		+	+	+	UL	Hpl	0.3	nc	CE	73.5	68.9	No
18	F	67	R		No			+	−	LL	Ad	0.3	nc	nc	67	41	Yes
19	F	61	P		No			+	+	UL	Ad	0.1	<0.1	CE	254.1	9.6	Yes
20	F	37	R		No		+	−	-	ectopic	Lymph node	nc	nc	CE	132.8	96.5	No

F: Female, M: Male, pHPT: Primary hyperparathyroidism, P-pHPT: Persistent primary hyperparathyroidism, R-pHPT: Recurrent primary hyperparathyroidism, MEN-1: Multiple endocrine neoplasia type 1, UR: Upper right, LR: Lower right, UL: Upper left, LL: Lower left, Hpl: parathyroid hyperplasia, Ad: parathyroid adenoma, CE: Cervical exploration, MIP: Minimally invasive parathyroidectomy, nc: not communicated, * persistent high PTH level with vitamin D deficiency (11.9 ng/mL).

**Table 3:** Comparisons between ^18^F-FCH PET/CT and 4D-CT, US, and parathyroid scintigraphy.

Analysis Results	^18^F-FCH PET/CT	4D-CT	*p* Value
Detection rate			
Lesion based	21/24 (87.5%)	15/24 (62.5%)	0.04
Patient based	26/37 (70.3%)	15/37 (40.5%)	<0.01
Sensitivity			
Lesion based	21/23 (91.3%)	15/23 (65.2%)	0.04
	^18^F-FCH PET/CT	US	*p* value
Detection rate			
Lesion based	7/7 (100%)	4/7 (57.1%)	0.25
Patient based	11/16 (68.7%)	4/16 (25%)	<0.05
Sensitivity			
Lesion based	6/6 (100%)	3/6 (50%)	0.25
	^18^F-FCH PET/CT	Parathyroid scintigraphy	*p* value
Detection rate			
Lesion based	13/15 (86.7%)	7/15 (46.7%)	0.08
Patient based	11/14 (78.6%)	7/14 (50%)	0.13
Sensitivity			
Lesion based	12/13 (92.3%)	6/13 (46.1%)	0.04

FCH: Fluorocholine, PET: Positron emission tomography, CT: Computed tomography, 4D: Four-dimensional, US: Neck ultrasound.

## Data Availability

Not applicable.
